# iBeacon Indoor Positioning Method Combined with Real-Time Anomaly Rate to Determine Weight Matrix

**DOI:** 10.3390/s21010120

**Published:** 2020-12-27

**Authors:** Yu Guo, Jiazhu Zheng, Weizhu Zhu, Guiqiu Xiang, Shaoning Di

**Affiliations:** Civil Engineering College, Nanjing Forestry University, Nanjing 210037, China; guoyu5186@njfu.edu.cn (Y.G.); paperpass@njfu.edu.cn (W.Z.); guiqiuxiang@njfu.edu.cn (G.X.); dsn17@njfu.edu.cn (S.D.)

**Keywords:** indoor positioning, iBeacon-based positioning, anomaly detection, isolation forest, Levenberg-Marquadt

## Abstract

This paper proposes an indoor positioning method based on iBeacon technology that combines anomaly detection and a weighted Levenberg-Marquadt (LM) algorithm. The proposed solution uses the isolation forest algorithm for anomaly detection on the collected Received Signal Strength Indicator (RSSI) data from different iBeacon base stations, and calculates the anomaly rate of each signal source while eliminating abnormal signals. Then, a weight matrix is set by using each anomaly ratio and the RSSI value after eliminating the abnormal signal. Finally, the constructed weight matrix and the weighted LM algorithm are combined to solve the positioning coordinates. An Android smartphone was used to verify the positioning method proposed in this paper in an indoor scene. This experimental scenario revealed an average positioning error of 1.540 m and a root mean square error (RMSE) of 1.748 m. A large majority (85.71%) of the positioning point errors were less than 3 m. Furthermore, the RMSE of the method proposed in this paper was, respectively, 38.69%, 36.60%, and 29.52% lower than the RMSE of three other methods used for comparison. The experimental results show that the iBeacon-based indoor positioning method proposed in this paper can improve the precision of indoor positioning and has strong practicability.

## 1. Introduction

Nowadays, Global Navigation Satellite System (GNSS) technology, navigation, and positioning services have become an indispensable service in people’s lives, especially for travel-related services. Such location services cannot be provided indoors, however, due to difficulties in receiving signals from GNSS satellites [1]. How to achieve fast, cheap, stable, and high-precision positioning and navigation in indoor contexts where satellite signals are missing has therefore become an urgent problem to be solved. At present, positioning and navigation services for indoor uses mainly rely on technologies such as Wireless Local Area Network (WLAN), Ultra-Wide Band (UWB), iBeacon, etc. [2,3,4]. Of these, iBeacon has been favored by many scholars due to its low cost, ease of operation, and signal stability [5]. In addition, indoor positioning and navigation services based on this technology have already been commercialized in many large shopping malls, parking lots, and other such venues [6].

Target positioning based on iBeacon technology mainly depends on the RSSI (Received Signal Strength Indication) value of the iBeacon base station broadcast signal [7]. Specifically, the current methods for solving positioning coordinates based on iBeacon technology are mainly divided into two types: fingerprinting technology and trilateration model. A comparison of these two main techniques is shown in Table 1 [8,9,10,11].

Fingerprinting technology has become a hot topic in current research due to its high stability and precision. The basic theory of Fingerprinting technology is to match the RSSI with the values in the database. It is not necessary to convert the RSSI value through a multi-mathematic model into a distance for positioning calculation. Therefore, this method does not require a distance estimation model and is more stable. This approach, however, requires a complete and detailed fingerprint database to be collected and constructed in the locations where it is used, alongside the installation of base stations. This in turn raises costs and increases the complexity of the positioning process, meaning that the technology cannot be easily applied in any location [12]. In contrast, the only preliminary work needed in the trilateration approach is the deployment of iBeacon base stations at fixed locations and determination of the ranging model parameters, meaning that it is simple and economical to implement.

On the other hand, the trilateration method cannot deliver highly precise positioning, with an absolute positioning error of about five meters [3]. How to improve the positioning precision and stability of the trilateration method has therefore become a problem worthy of research. The relative imprecision of the trilateration method arises partly from its reliance on a nonlinear positioning model, which seeks the best the optimal solution algorithm, and partly from the fact that environmental influences make the RSSI value of the iBeacon base station signal unstable. In addition, the particular trilateration model used also affects the positioning precision. At present, the most commonly used method is to perform differential linearization and then apply the least squares method to solve, or iteratively solve, the positioning coordinates [13,14].

In the above approach, as the RSSI is prone to fluctuations, it is often necessary to do some preprocessing of the original signal before positioning. At present, the main methods used for the preprocessing of abnormal RSSI signal values are filtering algorithms, such as Kalman filtering, particle filtering, mean filtering, etc. [15,16,17]. While these methods can often achieve good results when there is a large amount of RSSI signal data available, in practice it is often necessary to perform coordinate calculations within just a few seconds, which requires a signal anomaly detection method that can be applied effectively even when the amount of data is small. At the same time, most of the current preprocessing methods for RSSI signals only reduce noise and eliminate abnormal signals, and thus do not make full use of the abnormal characteristics of the signal source.

This paper therefore proposes the use of the isolation forest algorithm [18] to detect and eliminate signal anomalies even with a small sample size. It then calculates the anomaly rate of each signal source while removing abnormal signals, and sets the weight matrix using each anomaly rate and the RSSI after removing abnormal signals. Finally, using the most common trilateration model solution method—i.e., the least squares method—a weighted Levenberg-Marquardt (LM) algorithm [19] is constructed to achieve high-precision positioning indoors, based on iBeacon technology.

## 2. Materials and Methods

### 2.1. Overview

**Figure 1:**
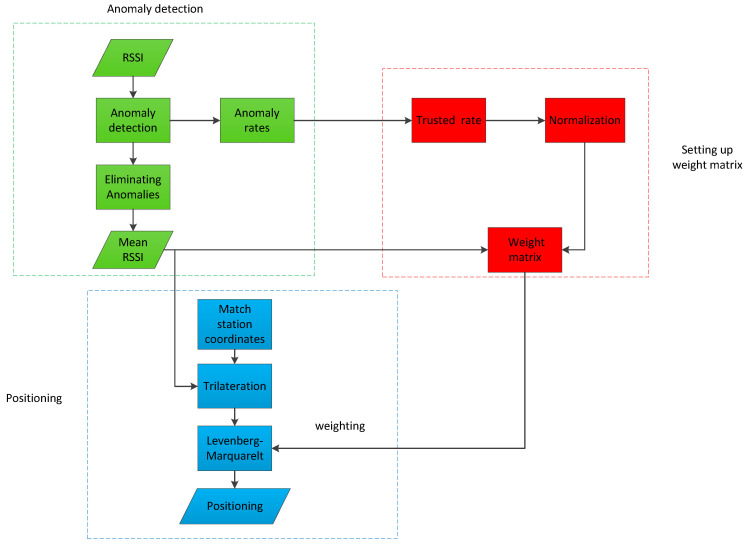
The workflow of anomaly detection and positioning.

The goal of this paper is to verify an indoor high-precision fast positioning method based on iBeacon technology. The basic theory of this paper is trilateration, applying the isolation forest algorithm and the weighted LM algorithm. The basic idea of this paper is to first collect the RSSI values of all iBeacon base stations deployed in the indoor scene, and then use the isolation forest algorithm to perform anomaly detection on the RSSI values of each signal source in a short period of time. After detecting abnormal signals, the anomaly rate of the signal broadcast by each iBeacon base station is calculated, and the average value of each group of signals, after removing the abnormal RSSI signal, is adopted as the final RSSI at the current point. Finally, a weight matrix is generated by combining the signal anomaly rate of each iBeacon base station with the RSSI value. Under the constraints of this weight matrix, the LM algorithm is used to solve the coordinates. The method flowchart of this paper is shown in Figure 1.

### 2.2. BLE-Based and Trilateration

**Figure 2:**
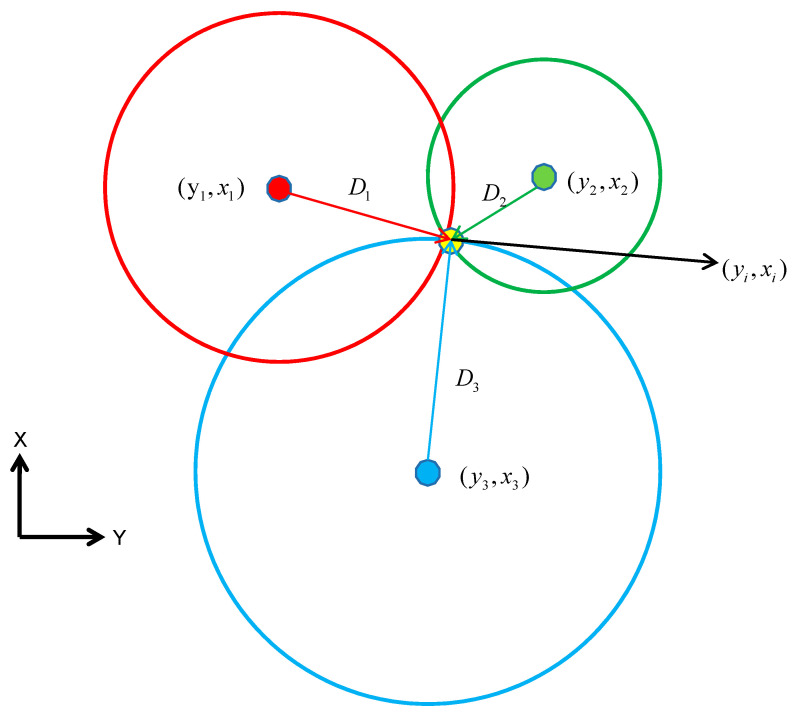
Trilateration using three iBeacons.

The trilateration method (see Figure 2) for indoor positioning based on Bluetooth low-energy devices is a fast and cheap positioning method. It determines the current point coordinates mainly by receiving the RSSI value broadcast by iBeacon base stations deployed in a fixed position in an indoor scene and applying a 2D plane coordinate solution model such as in Equation (1) below [20],

(1) $$\left\{ \begin{array}{l} {\left(x_{i}-x_{1}\right)}^{2}+{\left(y_{i}-y_{1}\right)}^{2}=D_{1}^{2}\\ {\left(x_{i}-x_{2}\right)}^{2}+{\left(y_{i}-y_{2}\right)}^{2}=D_{2}^{2}\\ \vdots \\ {\left(x_{i}-x_{n}\right)}^{2}+{\left(y_{i}-y_{n}\right)}^{2}=D_{n}^{2} \end{array}\right.$$

where $\left(x_{i},y_{i}\right)$ is the unknown coordinate of the current point, $\left(x_{n},y_{n}\right)$ is the fixed coordinate of each iBeacon base station, and $D_{n}$ is the distance between the current unknown point and each iBeacon base station. This distance can be calculated by an attenuation factor model between the RSSI value and *D*, as in Equation (2) [21]: Equation (1) only discusses the 2D situation, because the plane coordinates already satisfy most situations when used in daily life; however, Equation (1) can also be extended to 3D coordinates. At this time, we only need to add a Z coordinate (elevation coordinate) to the model. At this time, the basic formula in Equation (1) can be written as

$${\left(x_{i}-x_{n}\right)}^{2}+{\left(y_{i}-y_{n}\right)}^{2}+{\left(z_{i}-z_{n}\right)}^{2}=D_{n}^{2}$$

(2) $$P=P(d_{0})-10n\mathrm{lg}(\frac{d}{d_{0}})$$

where P is the RSSI value of the fixed iBeacon base station received at the current unknown point, $P(d_{0})$ is the RSSI value from the iBeacon base station at a certain distance, generally $d_{0}=1m$, and n is the signal attenuation factor in the current scene. In different environments, its value is also not always the same, and thus the relationship between the RSSI value and *D* (Distance between receiving signal point and base station) has to be obtained, as in Equation (3).

(3) $$D=10^{\frac{P(d_{0})-P}{10n}}$$

For the purposes of this paper, we set $P(d_{0})$ to $A_{n}$, and rewrite P to the collected RSSI value to get Equation (4). This means that there are then only two unknown parameters in the formula $A_{n},n$. These can be obtained by doing fixed distance experiments and fitting in the experimental data.

(4) $$D=10^{\frac{A_{n}-\mathrm{RSSI}}{10n}}$$

As for the solution of Equation (1), it is currently more common to linearize it first and then solve it by least squares, giving the final solution formula as Equation (5):

(5) $$X={\left(B^{T}WB\right)}^{-1}B^{T}Wl$$

where X is the current unknown point coordinates. B,l can be obtained by linearizing the Equation (1), and the weight matrix W is usually set $\frac{1}{{\mathrm{RSSI}}^{2}}$. When only the data broadcast by three iBeacons are used to calculate the current unknown point coordinates, the schematic diagram is shown in Figure 2.

Although it is relatively simple to use Equation (5) to calculate the coordinates, because the RSSI signal is affected by various environmental factors, there will be large signal fluctuations and thus low positioning reliability regardless of the distance from the iBeacon signal source. The weighting method in Equation (5) cannot reduce the error caused by this low credibility of the short-distance signal source. This paper therefore constructs a new weight matrix in Equation (5) according to the signal anomaly rates, and uses the nonlinear least squares LM algorithm to solve the positioning coordinates under the constraints of this weight matrix.

### 2.3. Anomaly Detection and Isolation Forest

The RSSI value of iBeacon’s Bluetooth signal is susceptible to fluctuations due to environmental interference during the collection process. To obtain high-precision positioning results, it is necessary to discover and eliminate abnormal RSSI in time. In other words, it is often necessary to eliminate outliers within a few seconds when performing indoor positioning and navigation. The relatively small amount of data available in these timescales makes the conventional robust estimation, Gaussian filtering, and other methods less effective [22].

This paper uses the isolation forest algorithm proposed by Zhou Zhihua et al. (2008) [18] for abnormal signal detection. This algorithm can achieve better anomaly detection results even with a small amount of data.

The basic idea of the isolation forest algorithm is to use several random planes to cut the data space repeatedly until there is only one data point in each data space. The specific process of anomaly detection is as follows [18].

(I) Randomly select ψ samples from all the data as the training subsample, and put it into the root node of the tree.

(II) Specify a dimension and randomly generate a plane cutting point Q within the range of the current node data. The range of the cutting point is between the maximum and minimum values of the training subsample.

(III) A plane is generated at the cutting point Q, and the data space of the current node is divided into two subspaces: put the points less than Q in the currently selected dimension on the left branch of the current node, and put the points greater than or equal to Q in the branch to the right of the current node.

(IV) Repeat steps II and III on the left and right branch nodes of the node, and continue to construct new leaf nodes until there is only one datum on the leaf node, or the tree has grown to the set maximum height.

(V) After constructing T isolation trees with the training subsamples, for each RSSI value in the entire data that needs anomaly detection, make it traverse the generated T isolation trees, and calculate the results in the T isolation trees. The average depth of each RSSI value is used to calculate the outlier of each RSSI value, where the outlier is defined as in Equation (6):

(6) $$S(\mathrm{RSSI})=2^{-\frac{E(h(\mathrm{RSSI}))}{c(\psi )}}$$

where E(h(RSSI)) is the average depth of a certain RSSI value in T isolation trees, and c(ψ) is the average value of the path length of the isolation tree generated by the training sample ψ.

(VI) Using Equation (6) and the isolation trees generated by training, calculate the S(RSSI) of the RSSI values broadcast by different iBeacons in turn. If the S(RSSI) is closer to 1, it is considered to be a signal outlier.

**Figure 3:**
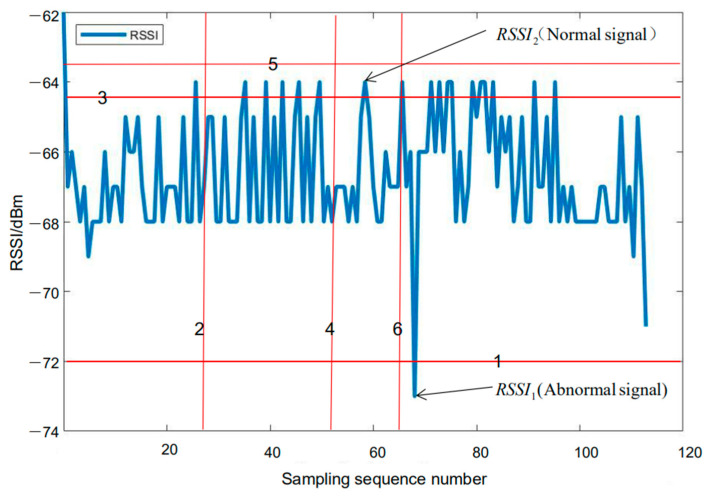
Isolation forest and spatial segmentation.

As shown in Figure 3, taking the RSSI value 0.2 m away from an iBeacon base station in the experimental scene as an example, ${\mathrm{RSSI}}_{1}$ only needs one plane cut to make it the only datum in the space, whereas ${\mathrm{RSSI}}_{2}$ needs to be cut six times to make it the only datum in the space. ${\mathrm{RSSI}}_{1}$ is therefore considered to be the abnormal signal value within that group of data. According to this principle, and combined with Equation (6), anomaly detection can be performed on all the collected RSSI signals. Of course this is just an ideal situation. Only one segmentation strategy was used to detect abnormal signals. In fact, a variety of segmentation strategies needs to be used in the experimental part, and finally the score is calculated according to Equation (6), which is used to determine whether a signal value should be defined as an outlier.

Compared with other methods, using the isolation forest algorithm to perform anomaly detection on the received RSSI has the following advantages. (1) The ability to perform anomaly detection unsupervised, thereby detecting anomalies in continuous data without prior data. (2) The amount of calculation is small, and distributed training and calculation can be realized well. (3) It has good effect and stability for data sets with small data volume and low data dimensions. This algorithm is therefore suitable for rapidly (i.e., within a few seconds) detecting abnormalities within the RSSI signals received from each iBeacon base station in an indoor location. Abnormally fluctuating signals can be detected in a relatively short time, and the abnormal rate of each signal source in that time period can be calculated. The equation for calculating the abnormal rate is as in Equation (7):

(7) $$\alpha =\frac{\mathrm{Abnormal}\;\mathrm{signals}}{\mathrm{All}\;\mathrm{signals}}$$

### 2.4. LM Optimization with Weighted Anomaly Rate

When using Equation (5) to solve the coordinates, if the equation is directly linearized, a certain error will be generated in the model. We therefore used the LM algorithm to solve the coordinates iteratively. From Equation (1), the plane coordinate solution model (8) and (9) can be obtained:

(8) $$f_{i}(Y,X)=\sqrt{{\left(Y-y_{i}\right)}^{2}+{\left(X-x_{i}\right)}^{2}}=D_{i}$$

(9) $$D_{i}-\sqrt{{\left(Y-y_{i}\right)}^{2}+{\left(X-x_{i}\right)}^{2}}=0$$

Furthermore, Equation (9) can be written as solving Equation (10):

(10) $$D_{i}-f_{i}(Y,X)=0$$

The LM algorithm is an improved Gauss-Newton method [23], and its iterative solution equation is as in Equation (11):

(11) $$x^{s+1}=x^{s}+\Delta$$

where $x^{s}$ is the plane coordinate vector ${\left[Y,X\right]}^{T}$ to be solved, $\left(x_{i},y_{i}\right)$ are the plane coordinates from every iBeacon base station involved in the coordinate solution, and $D_{i}$ is the spatial distance between the coordinate position to be solved and the iBeacon base station, which can be calculated by Equation (4). Furthermore, in order to find the optimal solution coordinates, the sum of squares $S(x)=\varepsilon^{T}\varepsilon$ of the residual vector $\varepsilon =D_{i}-f_{i}(Y^{*},X^{*})$ should be minimized, as shown in Equation (12).

(12) $$\left(Y^{*},X^{*})=\arg\min S(x\right)$$

The LM approach then performs a Taylor expansion of Equation (10) at $\left(Y,X)=(Y^{k},X^{k}\right)$, and omits terms of the second order and above so as to get the iterative formula of Equation (13):

(13) $$\Delta =-{\left(J_{f}^{T}J_{f}+\mu I\right)}^{-1}J_{f}^{T}\varepsilon^{f}$$

where $J_{f}$ is the Jacobian matrix of the function. For the i iBeacon base station signal sources, $J_{f}$ can be expressed as in Equation (14), I is the identity matrix of the coordinate vector to be solved, and $\varepsilon^{f}$ is the residual vector matrix of each iteration, which can be calculated as in Equation (15):

(14) $$J_{f}=\left[\begin{array}{l} \frac{\partial f_{1}}{\partial Y},\frac{\partial f_{1}}{\partial X}\\ \vdots \ddots \vdots \\ \frac{\partial f_{i}}{\partial Y},\frac{\partial f_{i}}{\partial X} \end{array}\right]$$

(15) $$\varepsilon (Y^{k},X^{k})=f_{i}(Y^{k},X^{k})-D_{i}$$

The μ in Equation (13), meanwhile, is the damping coefficient. When μ is small, the iteration process becomes the Gauss–Newton method of iteration, which has second-order convergence. When μ is large, on the other hand, the iteration process follows the gradient descent method, and the iteration converges quickly, the damping coefficient can be controlled by the Equation (16):

(16) $$\rho =\frac{f(x^{k})-f(x^{k}+h)}{L(0)-L(h)}$$

where h is the iteration step size, and L(0)−L(h) is as in Equation (17).

(17) $$L(0)-L(h)=-h^{T}J^{T}f-\frac{1}{2}h^{T}J^{T}Jh$$

In the iterative process, the damping coefficient μ is adjusted through ρ, then the current iterative solution process is selected. When the iteration terminates at $x^{s+1}-x^{s}<\lambda$, the coordinate solution is completed, and λ is a minimal value. The method for determining the initial damping coefficient of the iteration is $\mu_{0}=\max\{{\left({J_{f}}^{T}J_{f}\right)}_{ii}\}$.

It can be seen that the iterative solution of Equation (13) is not weighted, that is, the weight of the signal data of each iBeacon base station participating in the coordinate calculation is the same. In reality, however, the signal quality of different iBeacon base stations is different and thus the overall precision of the positioning can be improved by assigning different weights to iBeacon base stations. Accordingly, we add a weight matrix based on Equation (13) in combination with the anomaly detection results described in Section 2.3 to obtain Equation (18):

(18) $$\Delta =-{\left(J_{f}^{T}PJ_{f}+\mu I\right)}^{-1}J_{f}^{T}P\varepsilon^{f}$$

where the weight matrix is constructed by Equation (19):

(19) $$p_{i}=\frac{\beta_{i}}{{{\mathrm{RSSI}}_{i}}^{2}}$$

where ${\mathrm{RSSI}}_{i}$ is the mean value of the i-th iBeacon base station after removing the abnormal values within a period of time, and $\beta_{i}$ can be expressed by Equation (20):

(20) $$\beta =\mathrm{normalization}\{1-\alpha_{1},1-\alpha_{2}\ldots 1-\alpha_{i}\}$$

$\alpha_{i}$ can be obtained through Equation (7). According to Equation (20), the non-abnormal rate of each signal source is normalized. In other words, the iBeacon base stations at the location are sorted according to the fluctuations within a given period of time, and weights are assigned according to their abnormal rates. The optimal coordinates of the current point can be solved iteratively through Equations (11), (16) and (19) using the RSSI values of each iBeacon base station collected over that same period of time.

## 3. Experiment

The location for the experiment was an indoor venue with a size of around 92 m × 23 m. The experiment was mainly carried out in a corridor area, which had a minimum width of 2.4 m. We used a Xiao MI mi6 smartphone and its sensors for positioning. During our experiments, we held the smartphone horizontally in both steady and swaying states. The parameters and settings for the iBeacon base stations are shown in Table 2.

**Figure 4:**
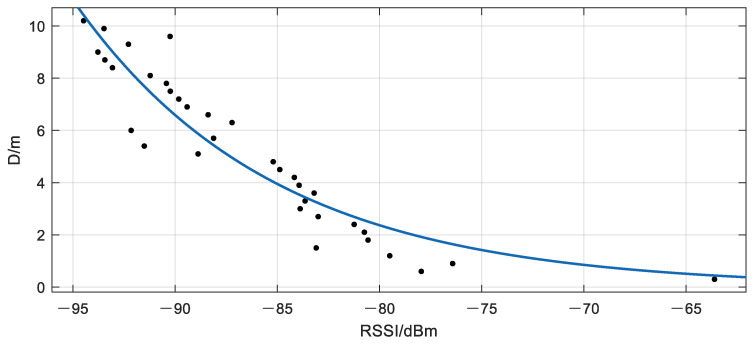
Model fitting.

**Figure 5:**
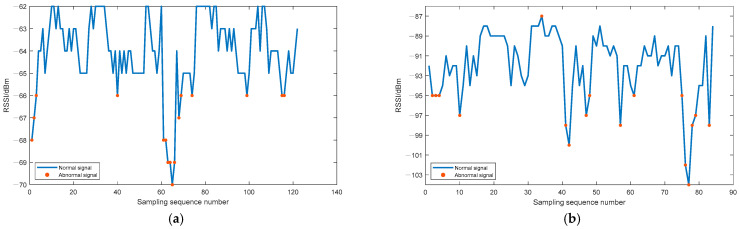
Anomaly detection: (**a**) Anomaly detection at 0.3 m. (**b**) Anomaly detection at 9 m

First, in order to obtain the specific values of A and n in the trilateration model, we set up an iBeacon base station at a fixed location in the test scene, and collected the RSSI values for a period of time at a fixed distance from the base station. Data were collected between 0.3 m and 10.2 m away from the base station, with an interval of 0.3 m. After using the isolation forest algorithm to detect anomalies and eliminate the abnormal signals, the signal strength values collected at each fixed distance were averaged in order to obtain the RSSI corresponding to the current distance. Then, the distance measurement model was fitted under the optimization of the nonlinear least squares algorithm (Figure 4). After identifying and eliminating the abnormal RSSI data at each fixed point, model fitting was performed, and the parameters $A_{n}$ and n in the ranging model were obtained by fitting with $A_{n}$ = −71.58 dBm, n = 2.647. It is worth noting that the fitting method used in this article is the least squares method. More specifically, it is the same as the method used in positioning solution: LM algorithm. As shown in Figure 4, this paper has achieved a good fitting effect based on Equation (4). Obviously, there are no data over fitting phenomenon. Besides, the anomaly detection results at 0.3 m and 9 m from the iBeacon station are shown in Figure 5. It can be clearly seen from Figure 5 that the signal anomaly rate at 9 m was significantly (1.54 times) higher than that at 0.3 m (the abnormal rate at 0.3 m is 13.11%, and that at 9 m is 20.23%). It was therefore necessary to perform real-time anomaly detection for each signal source in the process of positioning and solving.

**Figure 6:**
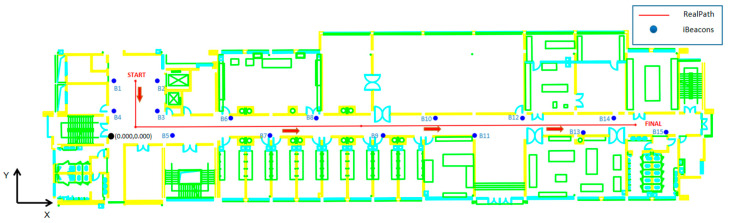
Collection path and iBeacons.

After obtaining the specific A and n parameters required by the ranging model, we set up iBeacon base stations with the same parameters in 15 fixed locations on the experimental floor. At the same time, we planned the path of the experimental data collection (Figure 6). In the subsequent coordinate calculation process, we performed coordinate calculations at intervals of 0.6 m, and used this to calculate and compare positioning errors.

After completing the iBeacon layout and data collection path planning, we carried out data collection experiments along the route shown in Figure 6. Then, the collected RSSI was matched with the MAC address, so that each corresponded to its signal source iBeacon base station. A custom coordinate system was used, the origin point for the coordinates is shown in Figure 6, and the MAC address and coordinates of each iBeacon base station are shown in Table 3.

**Figure 7:**
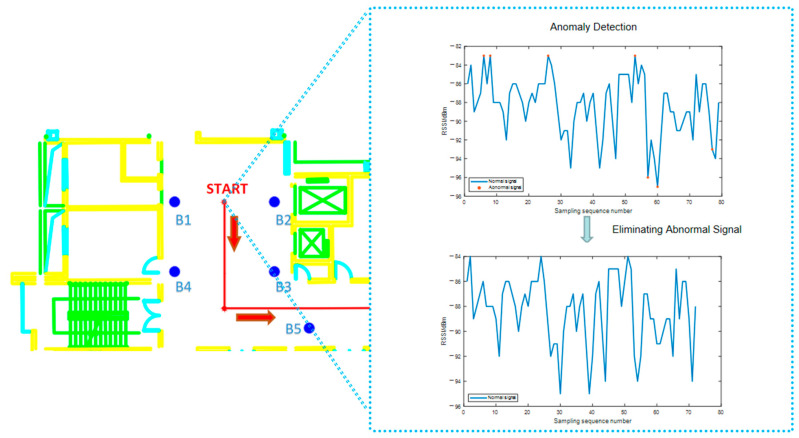
iBeacon “EB:CF:D1:9D:98:9F” RSSI at Start Point.

After preprocessing the collected data, i.e., performing the MAC and coordinate matching, we carried out anomaly detection on each group of data from different signal sources at 0.6 m steps according to the isolation forest algorithm described in Section 2.3. The anomaly rate of each group of data was calculated after eliminating the abnormal RSSI. For example, Figure 7 shows the original RSSI anomaly detection results received at the starting point from the iBeacon station B1 and the parallel data after the abnormal RSSI was eliminated.

**Figure 8:**
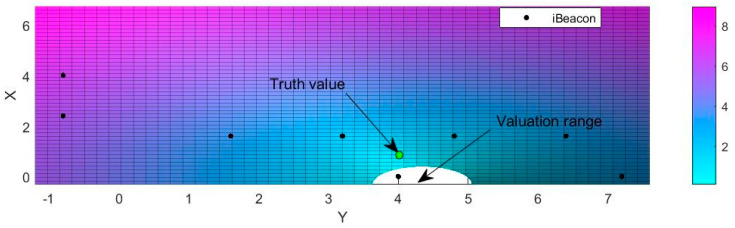
Coordinates solution.

Using the isolation forest algorithm for anomaly detection and calculating the anomaly ratio, the non-anomaly rate (trusted signal rate) was normalized and combined with the received RSSI to generate a weight matrix. Then, the weighted LM nonlinear least squares algorithm was used to calculate the coordinates of each point to be measured. The positioning results for an example point are shown in Figure 8. The valuation range in the figure refers to the coordinate area included in the 95% confidence interval. The coordinates at the center of that valuation range are chosen as the final positioning solution. As shown in Figure 8, the blue scale on the right represents the distance (m), and from small to high means an increase in distance.

**Figure 9:**
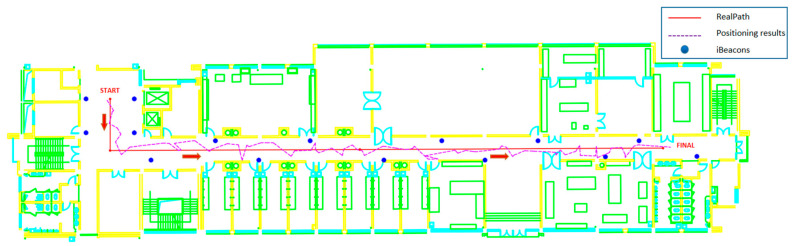
Positioning results.

According to the above method, the solutions for the 119 sets of data that were collected are shown in Figure 9. The error statistics are shown in Table 4.

It can be seen from the table that the maximum point position error is 3.527 m, the average value is 1.540 m, and the RMSE is 1.748 m. Among them, X, Y are the errors of the positioning results in the two vertical directions of the plane. About X, the Y direction as shown in Figure 6. The positioning accuracy obtained by the method proposed in this article is consistent with the current research status based on BLE technology (meter-level accuracy) [2], and is sufficient to meet the needs of indoor navigation and positioning. It is beneficial that the method proposed in this paper greatly improves the stability of positioning accuracy, which will be discussed in more detail in Part 4.

## 4. Discussion

**Figure 10:**
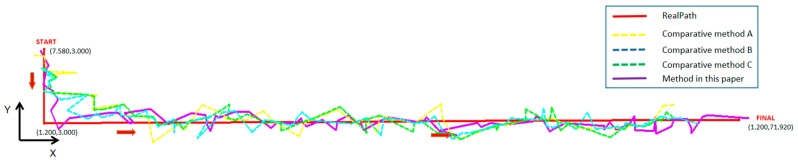
Positioning results for the comparator methods.

In order to better verify the effect of this method in reducing positioning errors, we also designed three comparison algorithms: (A) no anomaly detection together with a weightless LM algorithm to generate the solution [24]; (B) anomaly detection of signals to remove outliers together with a weightless LM algorithm to generate the solution; and (C) no anomaly detection, only the RSSI value used to set the weight matrix and then a weighted LM algorithm used generate the solution [25]. The respective errors of these three positioning methods are shown in Table 5. The experimental positioning results of the three algorithms are shown in Figure 10.

Comparing Figure 9 and Figure 10, it is obvious that the settlement results for individual positioning points have large fluctuations in all the comparator methods. This phenomenon will inevitably affect the user’s experience during the navigation phase, especially the excessive errors in the calculated coordinate values.

Except for Method C, the error in the X direction is worse than the error in the Y direction. This is because Method C only uses RSSI values for weighting; this will cause a certain degree of change in the direction of error distribution when solving. In fact, it is not difficult to see from Table 5. The error difference of the method proposed in this article in the two directions is also smaller than that of methods A and B. However, this does not affect the requirements for actual use.

From Figure 10, it can be seen that the result obtained by using the positioning method proposed in this paper is the closest to the real planned path. By comparing Table 4 and Table 5, it can be clearly seen that, compared to the three comparator positioning solution methods, the weighted LM algorithm combined with anomaly detection proposed in this paper can reduce errors and improve positioning precision, in both the X or Y direction, or on the plane. Compared with the A, B, and C comparator methods, the method proposed in this paper reduces the Mean YX error by 36.42%, 33.88%, and 26.60% respectively, and the RMSE by 38.69%, 36.60%, and 29.52%, respectively. In addition, the algorithm proposed in this paper reduces the maximum error by 46.64%, 46.50%, and 38.71% in respect to methods A, B, and C, respectively. This shows that the use of isolation forest algorithm for anomaly detection can effectively and quickly detect the abnormal fluctuations of the received signal. Eliminating these abnormal signals can reduce the positioning error and improve the positioning precision. What is more, weighting according to the abnormal ratio of each different signal source can further reduce the error in coordinate calculation.

**Figure 11:**
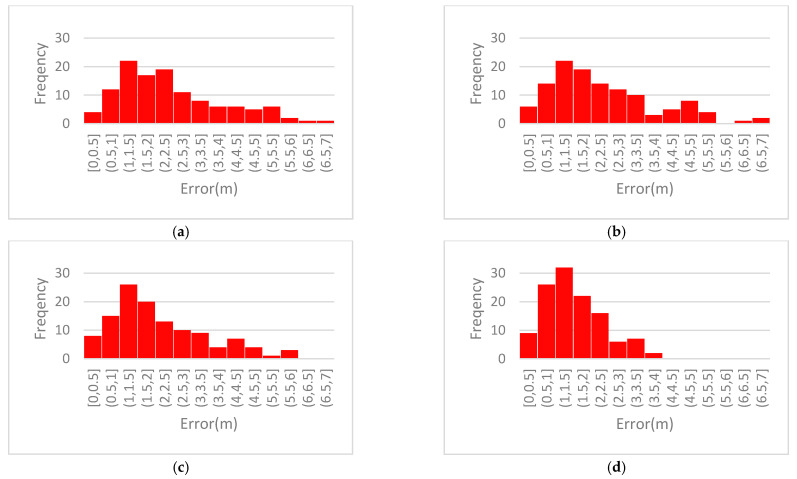
Error distribution frequencies: (**a**) Error frequency of A; (**b**) Error frequency of B; (**c**) Error frequency of C; (**d**) Error frequency of method in this paper.

**Figure 12:**
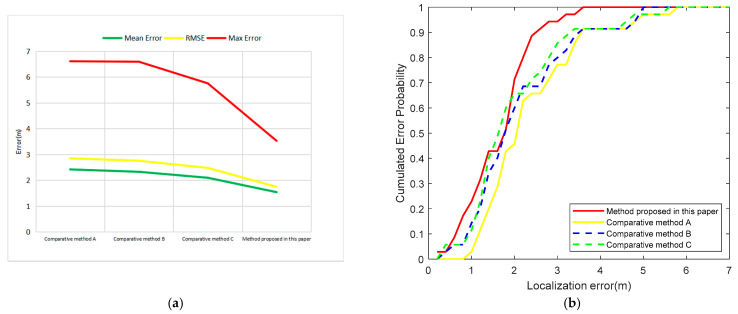
Error comparison discussion, (**a**) Error improvement; (**b**) Cumulative distribution functions of the localization error for the four methods.

In order further to illustrate the superiority of the positioning method proposed in this paper, the error distribution histogram (Figure 11) and the cumulative distribution curves (Figure 12) of the four positioning algorithms are shown.

From Figure 11, it can be easily seen that the error of the method in this paper is relatively concentrated and the dispersion is small. Seventy percent of the errors are distributed between 0.5 m and 2 m, and 85.71% of the points can ensure that the plane error is less than 3 m, which is obviously better than any of the other three comparative algorithms. It can be seen from Figure 12 that the method proposed in this paper has a very high degree of improvement compared with method A. Among them, compared with the intermediate methods B and C, eliminating the abnormal RSSI value also played a role to a certain extent; however, it is not obvious. The greatest improvement comes from weighting based on the real-time status of each signal source. This shows that using only the RSSI value for weighting is indeed easy to ignore the real-time signal fluctuations of the iBeacon station at a close distance. The method proposed in this paper can improve this phenomenon well. It can be seen from Figure 12b, meanwhile, that the cumulative distribution curve of this method is basically above the other curves, indicating that the error value aggregation is better than with the other methods. With the method proposed in this paper, the probability of an error being within 3.6 m is 1, that is, all errors are less than 3.6 m, which is obviously smaller than with any of the other three methods.

## 5. Conclusions

In this paper, we propose an indoor positioning method that detects anomalies in the RSSI of iBeacon signals, eliminates the detected RSSI anomaly, and then sets a weight matrix based on the detected anomaly rate and the RSSI. Under the constraints of this weight matrix, the LM algorithm is used for coordinate calculation. Using this method eliminates the need to establish a fingerprint database before application, and it can ensure lower error and higher positioning precision. Experiments prove that the average point error of the method proposed in this paper is 1.540 m and the RMSE is 1.748 m. Furthermore, in all coordinate points of the solution, the error can be controlled within 3.6 m, and in 85.71% of cases the point error is less than 3 m.

At the same time, in order to better compare the effects of the method proposed in this paper on error control and precision improvement, we have calculated the coordinate error of three other methods. Compared with using the LM algorithm to solve the coordinates without anomaly detection and without setting the weight matrix, the RMSE is reduced by 38.69%. Compared with only using the isolation forest algorithm to detect and eliminate the error, it is reduced by 36.60%, and compared to eliminating outliers but only using RSSI to construct the weight matrix, RMSE reduced by 29.52%. The mean error by the method proposed in this paper is also the lowest. This proves that the algorithm in this paper has the following advantages.

(1) Abnormal signal fluctuations can be detected in a short time, and the positioning coordinates can be solved in time according to the state of different signal sources, with good positioning precision.

(2) While ensuring the overall positioning precision, the loss of positioning accuracy caused by signal fluctuations is greatly improved.

## Figures and Tables

**Table 1:** Trilateration and fingerprinting.

Features	Trilateration	Fingerprinting
iBeacon collaboration	Yes	Yes
Fingerprint database and coordinate matching	No	Yes
Distance estimation mode	Yes	No
Implementation complexity and cost	Low	High
Stability	Low	High

**Table 2:** iBeacon base station settings.

Parameter	Setting
Size	39 mm × 39 mm
Time interval	100 ms
Coverage radius	80 m
Nominal signal at 1 m	−65 dBm
Battery life	2–3 years

**Table 3:** iBeacons’ MAC and Coordinates.

Number	MAC	Y	X
B1	EB:CF:D1:9D:98:9F	7.580	0.000
B2	FB:27:18:EB:98:F9	7.580	6.000
B3	C8:63:B7:72:11:B4	3.380	6.000
B4	EC:77:40:64:B5:6B	3.380	0.000
B5	EF:C8:4A:A0:29:E5	0.000	8.090
B6	FA:33:CD:CC:1D:DD	2.400	16.120
B7	FC:6A:5F:6B:4A:3C	0.000	21.520
B8	C4:FA:05:7F:81:CF	2.400	29.920
B9	DC:FC:82:05:CE:5E	0.000	37.120
B10	F9:46:D7:AA:7F:BE	2.400	44.320
B11	E1:69:1B:75:32:F4	0.000	49.720
B12	DC:29:58:B5:B4:53	2.400	56.320
B13	DE:0A:44:B5:84:C4	0.000	64.720
B14	C4:C5:B0:F9:2A:95	2.400	68.920
B15	F2:AE:CA:44:3F:30	0.000	76.120

**Table 4:** Errors in positioning using anomaly detection and weighted LM algorithm.

	Error (m)
Max Error S	3.527
Mean Error S	1.540
RMSE S	1.748
Max Error |Y|	2.346
Mean Error |Y|	0.579
RMSE Y	0.766
Max Error |X|	3.570
Mean Error |X|	1.290
RMSE X	1.571

S = $\sqrt{{\left(y_{p}-y_{r}\right)}^{2}+(x_{p}-x_{r})}$($\left(y_{p},x_{p}\right)$: positioning result, $\left(y_{r},x_{r}\right)$: real coordinate).

**Table 5:** Errors of comparative methods.

	Mthod in This Paper (m)	Error of A (m)	Error of B (m)	Error of C (m)
Max Error S	3.527	6.61	6.592	5.755
Mean Error S	1.540	2.422	2.329	2.098
RMSE S	1.748	2.851	2.757	2.480
Max Error |Y|	2.346	3.747	3.713	5.560
Mean Error |Y|	0.579	0.593	0.564	1.758
RMSE |Y|	0.766	0.902	0.865	2.193
Max Error |X|	3.570	6.480	6.570	4.916
Mean Error |X|	1.290	2.225	2.153	0.670
RMSE |X|	1.571	2.704	2.618	1.034

S = $\sqrt{{\left(y_{p}-y_{r}\right)}^{2}+(x_{p}-x_{r})}$($\left(y_{p},x_{p}\right)$: positioning result, $\left(y_{r},x_{r}\right)$: real coordinate).

## Data Availability

The data presented in this study are available on request from the corresponding author. The data are not publicly available due to privacy.
